# Neuroinflammation and Schizophrenia: New Therapeutic Strategies through Psychobiotics, Nanotechnology, and Artificial Intelligence (AI)

**DOI:** 10.3390/jpm14040391

**Published:** 2024-04-06

**Authors:** Freiser Eceomo Cruz Mosquera, Maria Camila Guevara-Montoya, Valentina Serna-Ramirez, Yamil Liscano

**Affiliations:** Grupo de Investigación en Salud Integral (GISI), Departamento Facultad de Salud, Universidad Santiago de Cali, Cali 760035, Colombia; freiser.cruz00@usc.edu.co (F.E.C.M.); maria.guevara03@usc.edu.co (M.C.G.-M.); valentina.serna02@usc.edu.co (V.S.-R.)

**Keywords:** psychobiotics, schizophrenia, nanotechnology, artificial intelligence, neuroinflammation

## Abstract

The prevalence of schizophrenia, affecting approximately 1% of the global population, underscores the urgency for innovative therapeutic strategies. Recent insights into the role of neuroinflammation, the gut–brain axis, and the microbiota in schizophrenia pathogenesis have paved the way for the exploration of psychobiotics as a novel treatment avenue. These interventions, targeting the gut microbiome, offer a promising approach to ameliorating psychiatric symptoms. Furthermore, advancements in artificial intelligence and nanotechnology are set to revolutionize psychobiotic development and application, promising to enhance their production, precision, and effectiveness. This interdisciplinary approach heralds a new era in schizophrenia management, potentially transforming patient outcomes and offering a beacon of hope for those afflicted by this complex disorder.

## 1. Introduction

Schizophrenia is a severe mental illness affecting approximately 1% of the global population. It is characterized by heterogeneous symptoms that can be divided into positive ones, such as delusions and hallucinations, and negative ones, like lack of motivation and social isolation [1,2]. Diagnosis is based on clinical observation of these symptoms and confirmed through psychiatric evaluations. Conventional treatments for schizophrenia primarily include antipsychotics, classified into three generations and mainly acting as antagonists of D2 dopamine and 5-HT2A serotonergic receptors [2,3]. However, these medications are effective in only about half of patients and can have serious side effects [2,4]. Therefore, the search for alternative treatment sources, such as psychobiotics, is of importance.

Emerging research in neuroinflammation has revealed its potential role in schizophrenia, leading to consideration of alternative treatments like psychobiotics [5,6,7]. These, being probiotics or prebiotics, could positively impact mental health by modulating the intestinal microbiota and, consequently, the neuroinflammatory pathways [7,8,9]. This approach arises from important questions about how the immune and neuroinflammatory systems interact and affect the neurobiology of schizophrenia. Recent studies have linked chronic neuroinflammation in the central nervous system (CNS) with schizophrenia, especially highlighting the relationship between schizophrenia and the expression of the major histocompatibility complex (MHC) on chromosome 6 [2,6,10]. This complex is found in brain and immune system cells related to adaptive immunity, such as CD19 and CD20B lymphocytes. Additionally, in untreated schizophrenic patients, an overstimulation of D3 dopamine receptors and an increase in the synthesis of interferon γ (IFNγ) in lymphocytes have been noted [6,11,12].

In schizophrenia, a variation in the intestinal microbiota has been observed compared to the normal microbial diversity of healthy individuals, highlighting an alteration in the abundance of certain bacteria and a decrease in others [7,13]. A direct relationship has also been found between the negative symptoms of schizophrenia and specific changes in the *Ruminococcaceae* family as well as an association between depressive symptoms and the Bacteroides genus. Intestinal dysbiosis in schizophrenia has also been linked to metabolic alterations induced by antipsychotic treatments such as risperidone and olanzapine [7,13].

As an alternative for the treatment of schizophrenia, the use of psychobiotics has been proposed [7]. These compounds, which include both probiotics and prebiotics specifically designed for psychiatric disorders, have a direct effect on the composition and function of the microbiota [14]. For instance, some psychobiotics can increase the abundance of short-chain-fatty-acid-producing bacteria, such as those of the Faecalibacterium genus, known for their anti-inflammatory properties, which could attenuate the neuroinflammation associated with schizophrenia. Moreover, these changes in the microbiota can influence the production of neurotransmitters such as Gamma-Aminobutyric Acid (GABA) and serotonin, directly in the intestine, which then impacts mood and behavior, offering a mechanism by which psychobiotics may mitigate both negative and depressive symptoms of schizophrenia [9]. The mechanisms through which psychobiotics modulate the microbiota include altering the intestinal pH to favor the growth of beneficial bacteria and inhibiting pathogens. Furthermore, some psychobiotics can act as prebiotics, providing specific substrates that encourage the growth of certain beneficial bacterial species, which, in turn, can contribute to the restoration of the intestinal microbiota balance [8,9]. This modulation of the intestinal environment not only supports digestive health but can also have systemic effects, including improving the intestinal barrier and reducing permeability, which is crucial for preventing the translocation of pro-inflammatory substances that could exacerbate the neuroinflammation observed in patients with schizophrenia [15].

Altering the intestinal microbiota with probiotics could improve symptoms of mental illnesses, establishing the importance of bidirectional communication between the gastrointestinal tract and the CNS through the microbiota–gut–brain axis (MGB Axis). Thus, anomalies in the intestinal microbiota have been associated with the onset of various neuropsychiatric disorders, including schizophrenia, highlighting intestinal dysbiosis as a distinctive feature of several neuropsychiatric diseases [8,13]. Neuroscientific studies have revealed that psychobiotics can modulate attention and vigilance towards negative emotional stimuli, as demonstrated in a functional magnetic resonance imaging study in healthy women who consumed a mix of probiotics (including *Bifidobacterium animalis* subsp. *Lactis* Bb12, and *Streptococcus thermophilus*, among others). A reduction in the activity of neural networks associated with emotional, somatosensory, and interoceptive processing was observed [7,8,9,13].

The integration of nanotechnology in the field of probiotics, prebiotics, and synbiotics offers innovative solutions to the challenges of their widespread use in the treatment and prevention of diseases like cancer and gastrointestinal disorders. Traditionally, the survival and efficacy of these nutraceutical compounds were limited by the adverse conditions of the human gastrointestinal tract, such as acidic pH and high oxygen concentration, which cause oxidative damage to the probiotic cells [16,17]. To ensure health benefits, a specific dose of viable cells is recommended, which often is not maintained until reaching the site of action due to these hostile conditions [16]. Nanotechnology emerges as a solution to these problems, allowing the development and characterization of nanomaterials and nanoparticles that encapsulate probiotics, prebiotics, and synbiotics—psychobiotics, in this particular case, for the treatment of schizophrenia—protecting them from adverse gastric conditions and ensuring their safe release at the site of action [9,17,18]. These nanomaterials can be of natural or synthetic origin and must be biocompatible, biodegradable, and generally recognized as safe (GRAS). The use of nanomaterials and nanostructures in the food industry not only improves food quality but also extends its shelf life, increases safety, and maintains cost and nutritional benefits [16,17,18].

The revolution of artificial intelligence (AI) in the field of psychobiotics represents a paradigm shift in the research and development of probiotic therapies [19]. AI not only assists in the identification of probiotics but also in their formulation to maximize their efficacy [19]. The application of techniques such as active machine learning to predict the effects of pharmaceutical excipients on the intestinal proliferation of probiotics is an example of how AI can improve the delivery and efficacy of probiotics in the organism. This approach allows a faster and more efficient optimization of psychobiotics, overcoming obstacles such as variability among individuals and adverse conditions of the upper gastrointestinal tract [19,20,21]. Tools like iProbiotics, which apply advanced machine learning techniques, are transforming the way therapies are discovered and optimized [20]. The study of the intestinal microbiome’s metabolome, crucial for understanding its impact on mental and physical health, has historically been challenging due to its complexity and variability. However, with the introduction of artificial models like ABIOME, a bioreactor imitation of the microbiota environment, and the implementation of algorithms like MARS (multivariate adaptive regression splines), it is possible to predict the metabolic activity of probiotic combinations and evaluate their therapeutic potential [21].

This multidisciplinary approach, combining advances in neuroscience, technology, and treatment, offers new perspectives and hopes for addressing schizophrenia, a complex and challenging mental illness. The aim of this study is to address how the convergence of psychobiotics, nanotechnology, and AI emerges as a promising therapeutic alternative, enhancing the development of personalized and effective solutions to improve the quality of life of people affected by this condition. This approach also lays the groundwork for a new era in mental health care, where personalization, technological innovation, and the holistic well-being of the patient are fundamental.

## 2. Materials and Methods

### 2.1. Search Strategy

Our study strategy was based on exhaustive searches across online databases, including PubMed, Scopus, ScienceDirect, Biomed Central, Google Scholar, and Web of Science, covering the period from 1 January 2024 to 15 February 2023 [22]. We employed specific keywords and their combinations, along with particular algorithms, to identify publications on the interaction between psychobiotics, nanotechnology, artificial intelligence (AI), and their impact on neuroinflammation associated with schizophrenia. This approach allowed us to refine the search results, focusing on topics directly relevant to the review article [23].

1. (Psychobiotics OR probiotics) AND schizophrenia AND neuroinflammation;
2. (Psychobiotics OR probiotics) AND schizophrenia AND (nanotechnology OR artificial intelligence).

### 2.2. Data Storage and Selection Tools

For the management of references and storage of relevant data, Zotero version 6.0.27 (accessed on 1 January 2024) was used. This software allowed for the efficient organization of the selected literature, facilitating the review process and selection of studies pertinent to the narrative review. Zotero was instrumental in cataloging sources, extracting important metadata, and maintaining an accessible record of all of the consulted literature.

## 3. Results

### 3.1. Schizophrenia: Definition, Symptoms, Risk Factors, Diagnosis, Classification, and Treatment

#### 3.1.1. Definition

Schizophrenia is a chronic and complex psychiatric disorder characterized by a wide range of psychotic, cognitive, and emotional symptoms [24]. The diagnostic criteria of the DSM-5 require the presence of at least two significant symptoms, such as delusions, hallucinations, disorganized speech, catatonic behavior, or negative symptoms for one month, with signs of the disorder persisting for six months [25]. It affects patients variably; more than 50% face long-term psychiatric problems, while about 20% suffer from chronic symptoms and disability. Moreover, schizophrenia is associated with reduced life expectancy, approximately 15 years less than the general population, and a suicide risk of 5% to 10% [24,25]. The reduction in life expectancy of individuals with schizophrenia can be attributed to factors such as suicide and the presence of concurrent medical and mental illnesses, including substance use disorders with prevalence rates up to 41%. Additionally, a chaotic lifestyle, an unhealthy diet, lack of physical activity, and the side effects of antipsychotic treatments contribute to the increased incidence of metabolic syndrome and cardiovascular and pulmonary diseases [26].

The etiology of schizophrenia is multifactorial, involving a complex interaction between genetic predispositions and environmental factors, such as complications during birth, prenatal infections, and psychosocial stress. These factors contribute to neurobiological alterations in the brain, including dysfunctions in the dopaminergic, glutamatergic, and serotonergic systems [25,26]. The clinical manifestations of schizophrenia are divided into three main categories: positive, negative, and cognitive symptoms. Positive symptoms include delusions and hallucinations, while negative symptoms refer to a reduction in emotional expression and avolition. Cognitive deficits, such as problems with working memory and attention, are also prevalent and significantly contribute to long-term disability [26,27]. To diagnose schizophrenia, a detailed clinical assessment, complemented by tests to rule out other conditions, is essential. The reliance on subjective criteria and the low specificity of clinical symptoms in early stages underscore the need for incorporating biological predictors [28,29]. Biomarkers, divided into peripheral (analyzed through blood samples) and central, offer insights into brain pathology. Specifically, genetic markers like the deletion in 22q11.2, linked to a significant risk of schizophrenia, highlight the role of genetic predisposition. Although identifying biomarkers for the prodromal phase remains a challenge, these could allow for early interventions and more personalized treatments, improving the prognosis [28]. Ongoing research on biomarkers is key to advancing the diagnosis and management of schizophrenia, towards more precise and effective medicine. Among the most relevant genetic markers is a deletion in the locus 22q11.2, implicated in approximately a quarter of schizophrenia cases and elevating the risk of developing the disease over a lifetime to up to 30% [28].

#### 3.1.2. Epidemiology

Evidence suggests that the pathogenesis of schizophrenia begins early in neurodevelopment, with perinatal complications, in utero adversities such as maternal infections and malnutrition, and obstetric complications. These early factors, along with markers of altered neurodevelopment and cognitive and motor deficits in childhood, predispose individuals to the development of schizophrenia in early adulthood. Moreover, cognitive deficits and negative symptoms, which often precede the first psychotic episode, significantly contribute to the morbidity of the disease [25]. The epidemiology of schizophrenia reveals a disorder impacted by various risk factors from neurodevelopment to environmental and socioeconomic influences [24,26,30]. Studies show global variability in the prevalence of schizophrenia, estimated at around 1% over a lifetime and schizoaffective disorder estimated at 0.32%, with incidence rates ranging between 7.7 and 43.0 per 100,000 individuals. The age of onset shows gender differences, occurring earlier in men (20–24 years) than in women (29–32 years) [31,32]. The proportions of individuals with the onset of schizophrenia spectrum disorders before the ages of 14, 18, and 25 are 3%, 12.3%, and 47.8%, respectively, with a peak at 20.5 years and a median age of onset at 25 years. The male/female ratio in disease burden has remained stable in the general population over the last 30 years [31,32]. This variability, along with temporal fluctuations in its incidence, contradicts the perception of a uniform risk. It highlights the relevance of factors such as prenatal stress, nutritional deficiencies, cannabis use, and adversities during childhood, underlining the weight of environmental conditions and early experiences in the development of the disease [24,26,32]. Advances in genetics and gene–environment interactions point towards new prevention strategies. The highest number of cases is found in Asia, while Sub-Saharan Africa and the Middle East present the lowest prevalence rates. The disease burden reflects a significant impact in low- and middle-income countries, demonstrating the need to improve research and prevention in these contexts [30,31,32]. In a study on schizophrenia conducted in the Valencia Region, Spain by Orrico-Sánchez et al. (2020) [33], during 2008–2015, individuals aged 15 to 64 years covered by the Regional Health System were examined. Following these subjects allowed for assessing the use of health services and determining the incidence of schizophrenia spectrum disorders, which was estimated at 6.2 per 1000 people, being higher in men than in women. A study by Osorio et al. (2019) in Caldas, Colombia, between 2010 and 2015, revealed a significant increase in the prevalence and incidence of schizophrenia. The prevalence increased from 0.03% to 0.11% and the incidence reached 70 cases per 100,000 inhabitants in 2015. This increase can be attributed to factors such as stress, unemployment, and social exclusion, as well as to greater access to pharmacological treatments. It is suggested to review the epidemiological profile of schizophrenia in other Colombian regions to identify the factors associated with its increase [34]. A study by Carteri et al. (2020) on schizophrenia in Brazil, between 2008 and 2019, reported an annual average of 154,009.67 hospital admissions and a cumulative incidence of 77.44 admissions per 100,000 inhabitants. The most admitted age groups were the elderly, followed by young adults. A general decreasing trend was observed, but an increase among youths also occurred, possibly related to the high prevalence of common mental disorders in teenagers, of which only 19.8% seek mental health services [35].

### 3.2. Neuroinflammation in Schizophrenia

Scientific research has begun to unravel the complexity of schizophrenia, a multifaceted psychiatric disorder, focusing recently on the interaction between neuroinflammation, gut dysbiosis, and its impact on the development and progression of the disease [10,11]. The interaction between immunological dysfunction and mental health opens new avenues for understanding schizophrenia beyond traditional neurochemical models, emphasizing the importance of considering the body’s inflammatory state and immune response as central factors in its development and manifestation [7,13,36].

**Figure 1:**
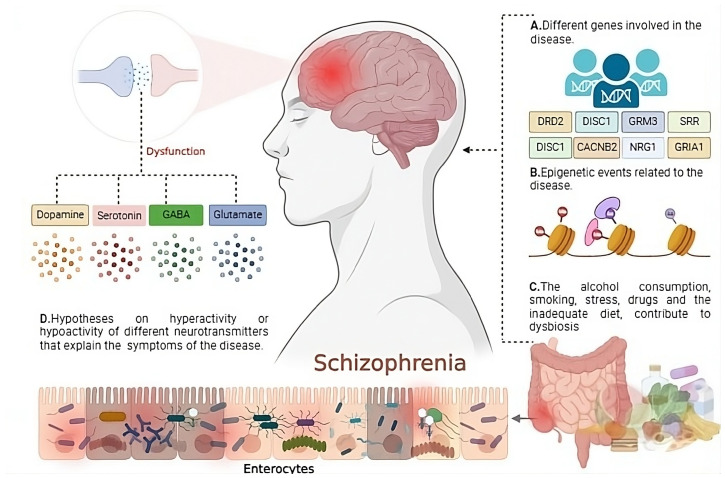
Diagram of neuroinflammation in schizophrenia. (**A**) Schizophrenia involves an interplay of genetic predispositions, with genes such as DRD2, DISC1, and GRM3 playing a role in its development. (**B**) Epigenetic modifications alter gene expression without changing the DNA sequence. (**C**) Environmental and lifestyle factors, such as alcohol consumption, smoking, stress, drug use, and diet can contribute to intestinal dysbiosis and, consequently, affect the immune and brain systems. (**D**) Hypotheses on the hyperactivity or hypoactivity of key neurotransmitters like dopamine, serotonin, GABA, and glutamate are considered central to explaining the symptomatology of schizophrenia. The figure was created using BioRender (https://www.biorender.com/) (accessed on 1 January 2024).

Neuroinflammation and dysbiosis, i.e., the imbalance in the composition of the gut microbiota, have been identified as potentially contributing factors to the pathology of schizophrenia [37]. This line of research is based on the growing understanding of how the gut microbiota affects not only gastrointestinal health but also brain function, through the gut–brain axis. Figure 1 illustrates the multifactorial nature of schizophrenia, highlighting the role of genetic, epigenetic, environmental, and neurochemical factors in its pathogenesis. Genes like DRD2, DISC1, and GRM3 are implicated in genetic predisposition, while epigenetic changes can influence gene expression without altering the DNA sequence. Environmental and lifestyle factors such as alcohol consumption, smoking, stress, drug use, and diet contribute to intestinal dysbiosis, impacting both the immune system and brain function. Central to the clinical presentation of schizophrenia are the theories suggesting hyperactivity or hypoactivity of neurotransmitters including dopamine, serotonin, GABA, and glutamate, which are critical to understanding the disorder’s symptoms [8,9,38].

This emerging paradigm suggests that modulation of the gut microbiome could offer new pathways for the treatment and prevention of psychiatric disorders like schizophrenia, highlighting the need to further explore the relationship between microbiota, immune response, and neurological function [10,11].

The constant interaction between the immune system and bacterial compounds in the gastrointestinal tract triggers immune responses that can contribute to various diseases [39,40]. Inflammatory responses have long been implicated in schizophrenia, though their origins are unclear. Schizophrenia is associated with elevated levels of IL-6, IL-8, and TNF-α and reductions in the anti-inflammatory IL-10. Additionally, elevated levels of antibodies against *Saccharomyces cerevisiae* (markers of intestinal inflammation) have been identified, consistent with gastrointestinal pathologies being one of the most common comorbidities of schizophrenia. Dysbiosis could exacerbate inflammation in the disorder through an increase in intestinal permeability [41].

Animal models have demonstrated how immune activation, especially maternal, can influence behavior and susceptibility to neuropsychiatric disorders in offspring. Having a dysbiotic microbiota has been identified as a significant factor in schizophrenia, suggesting that early interventions could prevent its manifestation [39]. The imbalance in microglial activation and the lack of polarization toward an anti-inflammatory state could contribute to neuronal impairment in schizophrenia. Research using animals has shown that alterations in microbiota, such as fecal microbiota transplantation from patients with schizophrenia, can induce disease-like behaviors, highlighting the importance of microbiota in the development of schizophrenia. While some antibiotic treatments have had negative effects, approaches targeting inflammation and microbiota have shown promising results, including the use of minocycline in animal models to protect against microglial activation, and suggest a link between microbiota and microglial activity in schizophrenia [39,40]. Microglia, making up 15% of all CNS cells, underline the cellular basis of neuroinflammation, activating in response to injury or disease. The “priming” of microglia via systemic inflammatory stimuli enhances their immune response to minor stimuli, highlighting the importance of stress-induced inflammation in psychiatric disorders. This phenomenon reinforces the vulnerability–stress–inflammation model, where physical and mental stress can trigger psychotic episodes in predisposed individuals [42].

A study by Iiopoulou et al., 2021 [43], investigates the relationship between dopamine, microglial activation, and neuroinflammation in schizophrenia, proposing that dopamine could have an immunomodulatory role in the brain. This analysis suggests that inflammation, primarily mediated by pro-inflammatory cytokines and peripheral immune cells, significantly contributes to the development of schizophrenia. Although elevated levels of pro-inflammatory cytokines have been observed in patients and an increase in inflammatory markers has been noted in postmortem studies, evidence on microglial activation, measured through positron emission tomography (PET) and Translocator Protein (TSPO), has shown varied results, including decreased or similar levels to controls. This study hypothesizes that dopamine modulation of microglia could influence neuroinflammation in a complex manner, affecting cytokine release and microglial activation. It highlights the possibility that different microglial populations respond variably to dopamine, depending on their activation phenotype and the dopamine receptors expressed. Furthermore, the research points to the need for deeper investigation into the relationship between dopamine and microglial activation in schizophrenia, suggesting that future studies should measure both TSPO and dopamine release to clarify the causal and region-specific link between them. This approach could improve our understanding of how microglia respond to alterations in dopamine levels and whether dopaminergic receptor dysfunction contributes to the pathology of schizophrenia [43].

Microglial activation and its interaction with dopamine underscore the role of inflammatory processes in the development of schizophrenia, with studies indicating an increase in pro-inflammatory markers and microglial density in patients’ brains. Research on inflammation as a biomarker in schizophrenia reveals its involvement in risk, development, manifestation, and treatment response, emphasizing the need for specific biological markers to address immune-related schizophrenia [39].

Patients with schizophrenia have been observed to have elevated serum antibody levels against fungal pathogens such as *S.cerevisiae* and *Candida albicans*, in addition to soluble CD14, a marker of bacterial translocation. These findings point to increased intestinal permeability, known as leaky gut, suggesting intestinal inflammation and could be an indicator of microbial dysbiosis. Pro-inflammatory cytokines can affect serotonin concentration through activation of the kynurenine pathway, reducing levels of tryptophan and serotonin and exacerbating symptoms of affective disorders. Compounds produced by certain bacteria can act as neurotoxins, interfering with the CNS’s elimination of ammonia and exacerbating neurological symptoms [7,44]. Kynurenate is a broad-spectrum antagonist of glutamate receptors, and N-Methyl-D-Aspartate (NMDAR) hypofunction is implicated in schizophrenia. Elevated levels of kynurenate have been observed in the postmortem brain tissue of individuals with schizophrenia, while rats treated with kynurenine-3-monooxygenase inhibitors have reduced prepulse inhibition, indicating the dopaminergic hyperactivity associated with schizophrenia [40,41].

In contrast, bacterial metabolites—particularly short-chain fatty acids (SCFAs) like butyric, acetic, and propionic acid, produced mainly by bacteria such as *Clostridium* spp., *Eubacterium* spp., and *Fusobacterium* spp.—have a crucial role in nourishing colonocytes, inhibiting pathogenic bacteria, and supporting the intestinal barrier [44]. The absence of these microorganisms can affect brain function, while their administration can improve microglial cell function and other aspects of mental and physical health [39].

The hypothalamic–pituitary–adrenal (HPA) axis and the enteric nervous system (ENS), also known as the “gut brain,” are essential in regulating stress and gastrointestinal activity, respectively. They interact closely with the gut microbiota, which includes neurotransmitter-producing bacteria like GABA, serotonin, dopamine, and norepinephrine. Microorganisms such as *Lactobacillus* spp., *Bifidobacterium* spp., and *Escherichia* spp. play a crucial role in the synthesis and metabolism of key neurotransmitters, including glutamic acid and serotonin, fundamental for learning, memory, and emotional health. The gut microbiota influences brain function by modulating neurotransmission and can indirectly impact the brain through the enteric nervous system and the production of neuroactive compounds. This complex bidirectional communication system underscores the significant role of microbiota in mental and physical health, pointing to the therapeutic application of probiotics and prebiotics in neuropsychiatric and gastrointestinal pathways [40,44].

To date, studies have been published investigating the differences in the microbiome between healthy controls and individuals with schizophrenia [41]. Mainly, an unchanged microbial diversity/richness was reported, but with significant differences in the abundance of specific taxa between groups. Interestingly, one study reported that, among those who were grouped with controls at baseline, 70% of patients experienced remission within 12 months, compared to only 28% with abnormal microbiota, an association that was not weakened by including the baseline GAF score as a covariate. However, there is considerable discordance among these results. At the phylum level, Proteobacteria and Firmicutes were found to be both significantly elevated and reduced in schizophrenia. This also occurs for taxa within the class Clostridia, although one study identified this class as a whole as being enriched in schizophrenia. Importantly, one of these studies investigated the oropharyngeal microbiome, which is structurally distinct from the gut microbiome. Perhaps the only consistently reported finding is a significant elevation of Lactobacilli in schizophrenia and people with an increased risk of schizophrenia, which even correlated with symptom severity. This is puzzling, considering that *Lactobacillus* spp. are common components of probiotics, thought to confer mental health benefits [37,40,41].

It is also necessary to recognize the dysregulation of the WNT/β-catenin pathway, important for cellular development, is associated with chronic neuroinflammation and oxidative stress in schizophrenia. This chronic inflammatory state, marked by alterations in cytokine secretion and abnormal activation of immunological receptors and neurotransmitters, contributes to neuronal dysfunction and oxidative stress, disrupting cellular communication and exacerbating schizophrenic symptoms. The interaction between neuroinflammation, oxidative stress, and the WNT/β-catenin pathway suggests an integrated pathogenesis model, opening the door to new therapeutic approaches based on the modulation of these processes. PPARγ agonists, which influence the WNT/β-catenin pathway and inflammatory processes, represent a potential therapeutic strategy, although more research is required to validate their efficacy and mechanism of action in the context of schizophrenia [6].

Genetically, the relationship between schizophrenia and variations in genes is related to immunity and inflammation—in particular, the complement system C4 gene, implying the activation of NF-κB by environmental factors. Analysis of postmortem brain tissue reinforces the presence of neuroinflammation in patients, correlating with acute psychotic symptoms and brain volume loss. Clinically, elevated levels of inflammatory markers suggest peripheral inflammation that varies with symptom severity, reflecting a bidirectional immune–brain connection [12].

Research suggests that chronic inflammation and prenatal or CNS development immune activation increase the risk of schizophrenia. This immune imbalance is reflected in the alteration of neurotransmitter systems and is implicated in the modulation of glutamatergic neurotransmission, essential in schizophrenia. Anti-inflammatory treatments such as COX-2 inhibition, minocycline, and acetylcysteine show potential in improving cognitive and negative symptoms in patient subgroups, suggesting inflammation as a viable therapeutic target [45,46,47].

Finally, the relationship between inflammation and changes in brain structure, explored through analysis including Mendelian randomization and gene expression correlation, highlights the association of IL-6 with alterations in brain structure, linking inflammation with developmental neuropsychiatric disorders. These findings point to inflammatory mechanisms as critical areas of research and potential targets for therapeutic interventions in schizophrenia [48].

A study by Williams et al., 2022 [48], investigated the relationship between inflammation and changes in brain structure in vivo, using multistage analysis that included Mendelian randomization (MR), gene expression correlation, and connectivity analysis. Using data from 20,688 participants from the UK Biobank and 6 postmortem brains from the Allen Human Brain Atlas (AHBA), the study focuses on genetic variants regulating the levels of interleukin 1 (IL-1), IL-2, IL-6, C-reactive protein (CRP), and brain-derived neurotrophic factor (BDNF). The results show that genetically predicted levels of IL-6 are associated with gray matter volume (GMV) and cortical thickness (CT) in specific brain areas, with no significant findings for IL-1, IL-2, CRP, or BDNF after correction for multiple comparisons. In the UK Biobank, IL-6 was associated with GMV in the medial, inferior, and fusiform temporal cortex, as well as with CT in the superior frontal region. The brain co-expression analysis in the AHBA sample revealed a highly interconnected network of genes preferentially expressed in the middle temporal gyrus (MTG), forming a protein–protein interaction network with IL-6. Genes differentially expressed in MTG were functionally enriched for biological processes related to schizophrenia, autism spectrum disorder, and epilepsy. This study suggests that genetically determined IL-6 is associated with brain structure and could affect areas involved in developmental neuropsychiatric disorders, such as schizophrenia and autism.

Manipulation of the gut microbiota through probiotics is postulated as a method to regulate immune responses and improve both psychiatric symptoms and gastrointestinal comorbidities in patients with schizophrenia [36,39,44]. This interaction highlights how intestinal dysbiosis and the immune approach can reveal the pathogenesis of schizophrenia, suggesting the use of antipsychotics and psychobiotics as innovative therapeutic strategies [7,49].

The exploration of neuroinflammation in schizophrenia highlights a significant shift towards understanding the disorder beyond conventional neurochemical models, focusing on the interplay between neuroinflammation, gut dysbiosis, and immunological dysfunction. This emerging perspective posits that the body’s inflammatory state and immune response play central roles in the development and manifestation of schizophrenia.

### 3.3. Gut Microbiota and Schizophrenia

The transition from unicellular to multicellular organisms approximately 3 to 3.5 billion years ago marked the beginning of a co-evolution between microbes and humans, resulting in an interconnected physiology that influences the evolving phenotypes of all life forms [50]. The gut microbiota plays crucial roles in the organism, including maintaining proper intestinal function, digesting food through the secretion of digestive enzymes, and converting complex nutrients into simpler organic compounds. Moreover, it participates in the absorption of digested food and in the synthesis of vitamins, especially B-group vitamins. Intestinal microorganisms produce SCFAs through the anaerobic fermentation of indigestible carbohydrates, with butyric acid being particularly important for the nutrition and growth of colon epithelial cells [40,50].

The gut microbiota also plays a role in neutralizing toxins and carcinogenic compounds, creating the intestinal barrier, and protecting against pathogens. Additionally, it has immunomodulatory functions, regulates cytokine levels, and interacts with the lymphatic tissue of the digestive tract, constituting the largest lymphatic organ in the human body. Alterations in the quantity and composition of the gut microbiota (intestinal dysbiosis) can lead to anomalies such as disorders in peristalsis, digestion, absorption, vitamin production, metabolism, and in the function of the intestinal barrier, as well as excessive stimulation of the immune system [37,41,49].

Studies using research models have explored the impact of gut microbiota on brain development and function, including the use of germ-free mice, probiotic and antibiotic therapies, fecal microbiota transplants, and research on infections [44]. The key concept of the gut microbiome is linked to the CNS—how intestinal dysbiosis and the immune approach could unveil the pathogenesis of schizophrenia, discussing the use of antipsychotics and psychobiotics to develop new therapeutic strategies [7,13].

Immunological mechanisms suggest an inflammatory basis for psychiatric disorders, where anti-inflammatory cytokines affect neurohormonal and neurochemical functions. Increased intestinal permeability, known as leaky gut syndrome (LGS), plays a major role in the systemic inflammatory response, caused by intestinal dysbiosis, damage to enterocytes, and stress, contributing to the pathophysiology of depression. This condition facilitates bacterial translocation, especially of Gram-negative bacteria containing lipopolysaccharide (LPS), triggering an overactivation of the immune system and an increase in pro-inflammatory cytokines that negatively affect the CNS [7,37,49]. Intestinal dysbiosis is associated with numerous intestinal and systemic diseases, such as intestinal candidiasis, Crohn’s disease, ulcerative colitis, pseudomembranous enteritis, inflammatory bowel disease, food allergies, intolerances, and colorectal cancer [50].

Moreover, it is observed that pro-inflammatory cytokines affect serotonin concentration by activating the kynurenine pathway, which decreases the levels of tryptophan and serotonin, exacerbating the symptoms of affective disorders. Bacteria such as Clostridium, Proteus, and Enterobacteriaceae, by decomposing proteins, produce ammonia, a neurotoxin that interferes with the CNS’s elimination of ammonia, exacerbating neurological symptoms [50].

Modern research on schizophrenia reveals a complex interaction between genetic, epigenetic factors, alterations of the gut microbiome, immune system anomalies, and environmental factors, yet without identifying a single cause and definitive mechanism [51,52,53].

Current research suggests that the gut microbiota plays a crucial role in the pathogenesis and manifestation of psychotic disorders, with risk factors extending from prenatal life to adolescence. During prenatal life, maternal infections such as Toxoplasma gondii, severe maternal stress, and malnutrition are linked to an increased risk of schizophrenia, affecting the gut microbiota and its function. Studies demonstrate that certain bacteria, such as Bacteroides fragilis, can mitigate behavioral deficits and correct dysfunctions in intestinal permeability induced by these factors [50].

In early life, obstetric complications, infections, and early stress are factors that alter the gut microbiota, influencing neurological development and increasing the risk of psychotic symptoms [50]. The gut microbiota in the first days of life is unstable and of low diversity, influenced by multiple maternal sources. Microbial diversity increases during the first three to four years of life, influenced by the mode of birth, feeding, and early use of antibiotics. Early postnatal brain development coincides with the acquisition and reorganization of the gut microbiome, suggesting an association between the composition of the infant microbiome and communication, personal, social, and fine motor skills at three years of age [50].

The mode of delivery, including cesarean births, alters the gut microbiota and is associated with risks of asthma, allergies, and obesity in offspring. Obstetric complications have been linked to schizophrenia, although the relationship between cesarean sections and schizophrenia is tenuous (see Table 1) [44,54,55]. Feeding (breastfeeding versus formula), early use of antibiotics, and malnutrition are also important factors shaping the gut microbiome [50]. This complicates the identification of diagnostic markers and the development of effective antipsychotic medications. A significant advancement is the discovery of the alteration of the gut microbiome in patients with schizophrenia and how it communicates with the brain through the gut–brain axis, implicating tryptophan metabolism, neurotransmitter synthesis, and immunoregulatory pathways [53,56]. Adolescence presents its own risks with smoking, drug use, and exposure to urban environments and migration, all associated with an increased risk of psychotic disorders and changes in the gut microbiota. These factors can decrease the diversity of the microbiota, negatively impacting mental health [50].

Stress and disruptions in the circadian clock system have a profound impact on the composition of the gut microbiota (GM), showing how mental well-being and biological rhythms play a crucial role in digestive health. Stress can reduce the number of beneficial species such as *Lactobacillus* spp. and *Bifidobacterium* spp., while increasing pathogenic and non-pathogenic strains of *E. coli* and species of the genus *Clostridium* spp. This alteration not only decreases the microbiota’s capacity to support healthy metabolic and immunological functions but also increases vulnerability to infections and intestinal diseases. On the other hand, the circadian clock system, responsible for regulating daily rhythms in our body, affects the diurnal fluctuations of the GM. Desynchronization between our life cycles and this internal biological clock, whether due to chronic stress or changes in sleep patterns, can cause dysfunction in the gut microbiota, resulting in a decline in beneficial bacteria such as *Lactobacillus* spp. and an increase in pathogenic bacteria, highlighting the interconnection between the circadian rhythm, stress, and intestinal health [54,55].

Furthermore, occupational and environmental exposure to contaminants, as well as variations in diet, exert a significant influence on the composition of the GM, highlighting the importance of external factors in our digestive health. Contaminants such as heavy metals, pesticides, and PAHs can modify the composition of the GM, while shift work and exposure to certain work environments can alter the microbiota, indicating possible health risks. On the other hand, diet plays a fundamental role in the diversity and abundance of the GM, influencing metabolism and immune responses. Dietary fibers, fermented by the GM to produce SCFAs, are beneficial for colon health. Variations in diet, such as Mediterranean, ketogenic, vegetarian, or vegan diets, have significant impacts on the composition of the GM, underlining how our eating habits can directly shape our intestinal ecosystem and, by extension, our overall health [44,54,55].

In schizophrenia, consistent alterations in beta diversity and changes in the abundance of certain taxa such as Ruminococcus, Roseburia, Veillonella, Proteobacteria, Fusobacteria, in addition to variations in *Aerococcaceae*, *Bifidobacteriaceae* (among others), are influenced by factors such as diet, smoking, antipsychotic medication, and age [62].

Understanding how microbes communicate with the brain has opened new avenues for exploring brain development and neuropsychiatric disorders, especially in cases like schizophrenia. Significant advancements in preclinical research have shown that neurotransmission, neurogenesis, myelination, dendrite formation, and the organization of the blood–brain barrier are influenced by the microbiota–gut–brain (MGB) axis. Behaviorally, this axis modulates cognitive function, social interaction, locomotor activity, and stress management [50].

Among the interconnected mechanisms of the MGB axis is the immune system, which allows microbes to influence brain immunoregulation; tryptophan metabolism, crucial for serotonin synthesis and brain function; the HPA axis, involved in stress response; the vagus nerve, acting as a direct communication route between the gut and the brain; and microbial metabolites, such as SCFA, which modulate brain function through energy and neuroprotective effects [63].

Communication pathways between the gut microbiota and the brain include the vagus nerve, a principal component of the parasympathetic nervous system. This nerve, with 80% afferent fibers and 20% efferent fibers, does not directly traverse the epithelial layer of the digestive tract, implying that communication with the luminal intestinal microbiota is indirect. Enteroendocrine cells, expressing Toll-like receptors (TLRs) for microorganism detection, can influence the vagus nerve through the release of serotonin and intestinal hormones, thus regulating gastrointestinal motility, secretion, and food intake [64].

Recent studies have shown that both unmedicated and medicated patients with schizophrenia exhibit alterations in the diversity and composition of their gut microbiota, related to the severity of the disease. It is suggested that immunomodulation and alterations in the gut–brain axis play a key role in the development of schizophrenia. Intervention studies using probiotics have shown promising results, including the reduction of antibodies against *C. albicans* and the improvement of psychiatric symptoms, suggesting that probiotic treatment could be a potential strategy to improve the symptoms of schizophrenia. Animal models have provided strong, albeit indirect, evidence of the role of the gut microbiota in psychiatric symptomatology, demonstrating that stress affects both the function and composition of the gut microbiota and the host’s metabolism. Modifying the gut microbiota through fecal microbiota transplant (FMT) can directly moderate metabolic function, causing behavioral changes in both rodents and humans, and suggests that microbiota-induced alterations in neural signs and tryptophan metabolism could secondarily affect physiology and brain function [62].

Studies on the oropharyngeal microbiota in schizophrenia have highlighted significant differences in microbial composition between individuals with schizophrenia and controls, with a higher proportion of Firmicutes in patients and greater species diversity in controls. Lactic bacteria, including *Lactobacillus* spp. and *Bifidobacterium* spp., were more abundant in individuals with schizophrenia, with *Lactobacillus gasseri* particularly prominent. Additionally, differences were identified in metabolic pathways, with some enriched and others diminished in schizophrenia, suggesting a link between the oropharyngeal microbiota and schizophrenia [62,65].

The involvement of the gut microbiome in schizophrenia is evidenced by the presence of autoimmune and gastrointestinal comorbidities, low-level inflammation, and increased intestinal permeability. Intestinal dysbiosis appears to play a central role in the pathogenesis of schizophrenia, with alterations in tryptophan metabolism and neurotransmitters suggesting a connection between dysbiosis and mental disorders. Studies point to a causal relationship between dysbiosis and schizophrenia, affecting specific metabolic pathways and underscoring the potential of the gut microbiome as a therapeutic target [7,37,66].

The diversity of the microbiome in individuals with schizophrenia shows significant differences in the composition and abundance of certain taxa between patients and controls. These changes are associated with clinical features of schizophrenia, including symptom severity and overall functioning, although the underlying mechanisms are not yet fully understood [62]. Probiotic supplementation has offered inconsistent results, indicating the need for more research to understand how certain bacteria influence the development of schizophrenia [52,53].

The Human Microbiome Project has revealed the complexity and individualization of the gut microbiome, influenced by hereditary, dietary, and environmental factors. Schizophrenia, as a disorder, may be affected by various factors, including aging and early life events, as well as lifestyle and treatment with antipsychotics, highlighting the importance of a deeper understanding of how these factors interact with the gut microbiome [62].

The burgeoning field of research linking the gut microbiota to schizophrenia underscores the intricate relationship between our intestinal ecosystem and brain health. Studies have consistently shown significant alterations in the microbial diversity and composition of individuals with schizophrenia compared to healthy controls, implicating factors such as diet, medication, and lifestyle in these differences. The gut–brain axis emerges as a pivotal pathway through which the microbiota influences neurological development, immune function, and even the severity of psychiatric symptoms. This axis, modulating essential aspects of neurotransmission, neurogenesis, and behavioral responses, opens promising avenues for novel therapeutic strategies. Probiotic interventions, in particular, demonstrate potential in mitigating psychiatric symptoms and improving overall mental health.

### 3.4. Impact of Blood–Brain Barrier Dysfunction and Microbiota on Schizophrenia

The blood–brain barrier (BBB), a crucial structure that protects the CNS from potentially harmful substances in the blood, plays a significant role in the pathogenesis of psychotic disorders such as schizophrenia. Dysfunction of the BBB allows the passage of inflammatory molecules and alters glutamate homeostasis, contributing to neuronal and synaptic dysfunction. Preclinical research has shown that the gut microbiota can influence the integrity of the BBB, modulating the expression of tight junction proteins and affecting its permeability. The suppression of specific proteins, such as claudin-5, has been linked to learning deficits and anxious behaviors, underscoring the connection between the BBB and schizophrenia [40,50].

The relationship between the gastrointestinal tract and the brain, originating from the same part of the embryo, highlights the importance of the gut microbiota and the BBB in neurological development. The gut microbiota affects the permeability of the BBB even before birth, with germ-free (GF) mice showing increased permeability due to reduced expression of occludin and claudin-5. This interaction suggests that the gut microbiota plays a critical role in maintaining the integrity of the BBB and, by extension, in neurological health [67,68].

Stress factors, both prenatal and in early life stages, have a notable impact on the gut microbiota and, consequently, on the function of the BBB. Alterations in the microbiota due to stress can lead to changes in stress responses and social behavior in animal models, indicating a link between the microbiota, the BBB, and schizophrenia. The dopaminergic and glutamatergic hypothesis of schizophrenia is reinforced by evidence showing the influence of the microbiota on dopaminergic and glutamatergic neurotransmission, offering a new paradigm for understanding and treating this disorder [50,67].

Research on schizophrenia has also identified various risk factors, from prenatal life to adolescence, that interact with the gut microbiota, suggesting a significant role of the latter in the manifestation of psychotic disorders. From maternal infections and prenatal stress to smoking and urban environment in adolescence, these factors can influence the composition of the gut microbiota, affecting the integrity of the BBB and contributing to the development of psychotic disorders [50,68].

### 3.5. The Role of Brain-Derived Neurotrophic Factor in Schizophrenia

Brain-derived neurotrophic factor (BDNF) is an essential protein that plays a crucial role in neurodevelopment and in the regulation of fundamental neural processes, such as learning and memory [69]. This neurotrophic factor is not only important for the survival of existing neurons but also promotes the growth and differentiation of new neurons and synapses. Given its importance in neuroplasticity and cognitive function, BDNF has become a focus of interest in psychiatric disorder research, especially schizophrenia [41,70].

In neurodevelopmental models of schizophrenia, alterations in BDNF levels are a recurring theme, suggesting a link between the characteristic cognitive dysfunction of this disease and the brain’s reduced capacity to adapt and respond to the environment. Studies have shown that individuals with schizophrenia often exhibit reduced BDNF levels in the brain, which could contribute to deficiencies in forming new memories, learning, and the ability to perform tasks requiring cognitive flexibility and adaptation. The reduction in BDNF levels in schizophrenia is associated with a decrease in neuroplasticity, meaning a lesser ability to form and reorganize synaptic connections in response to new information or experiences. This limitation in brain plasticity could be one of the fundamental reasons behind the cognitive deficits observed in patients with schizophrenia, such as problems with abstract thinking, attention, working memory, and executive function [11,70,71].

Furthermore, research has suggested that stress, one of the environmental factors associated with the development and exacerbation of schizophrenia, can have a negative impact on BDNF levels. Chronic stress has been shown to reduce BDNF expression, which could worsen or precipitate cognitive dysfunction in individuals predisposed to schizophrenia. This highlights the importance of considering both genetic and environmental factors in the study of how BDNF affects the pathogenesis of schizophrenia [11,70,71].

Mutations in the BDNF gene have been associated with hyperphagia and obesity, while exogenous administration of BDNF has been shown to induce weight loss in animal models [41]. Obesity is highlighted as one of the most relevant and increasing independent risk factors for cardiovascular disease (CVD), leading to higher morbidity, mortality, and reduced life expectancy. Recently, obesity has also been associated with a low-grade inflammatory state. However, these findings do not fully explain the clinical situation of obesity, and new mechanisms responsible for its development and progression are currently being investigated. Factors involved in energy expenditure balance, lipid and glucose control, and cardiovascular homeostasis are now considered as a new family called metabotropic factors, including nerve growth factor (NGF) and BDNF [72]. BDNF, synthesized not only in neurons but also in immune cells, adipocytes, endothelial cells, and monocytes, plays a multifaceted role from its neurotrophic activity to its involvement in inflammation, metabolism, and cardiovascular diseases. This has led to the coining of the term “triactome,” explaining the close interactions between the brain, the immune system, and adipose tissue in the development of cardiometabolic diseases [72,73].

The complicated relationship between schizophrenia, obesity, and the microbiome is hampered by the lack of data indicating causal relationships. Preliminary evidence suggests that supplementation with probiotics could benefit people with schizophrenia in terms of symptoms and comorbid conditions, despite the apparent lack of effect on the core aspects of the disorder. Larger-scale studies with consistency in treatment options and measured outcomes are needed [41].

### 3.6. Psychobiotics in the Treatment of Schizophrenia

**Figure 2:**
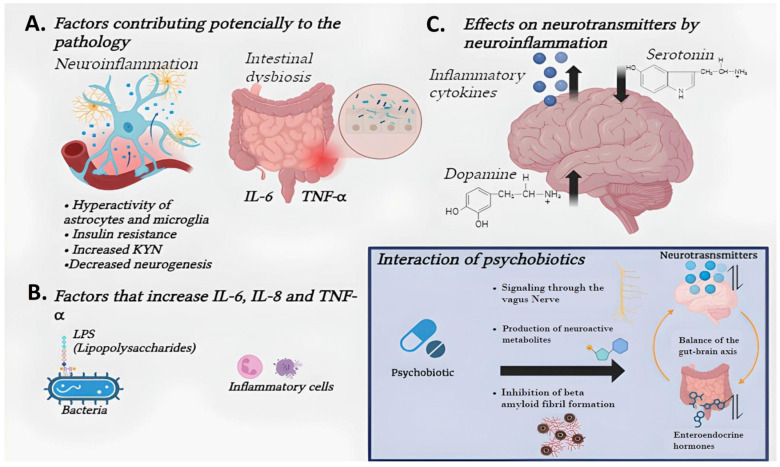
The role of psychobiotics in neuroinflammation in schizophrenia: (**A**). Factors contributing to potential pathology include the hyperactivity of astrocytes and microglia, increased levels of inflammatory cytokines like IL-6 and TNF-α, and reduced neurogenesis. (**B**). Contributors to the elevation of cytokines such as IL-6, IL-8, and TNF-α include bacterial lipopolysaccharides (LPS), signaling the involvement of microbial elements in neuroinflammation. (**C**). Neuroinflammation’s effects on neurotransmitters illustrate how inflammatory cytokines can disrupt the balance of critical neurotransmitters, such as dopamine and serotonin. Thus, the role of psychobiotics extends beyond mere microbiota modulation, as they are instrumental in augmenting neurotransmitter levels, fortifying the integrity of the gut–brain axis, and orchestrating the activity of enteroendocrine hormones. By doing so, they unveil a promising and multifaceted therapeutic avenue for the management of schizophrenia, offering a glimpse into future treatments that harness the symbiotic relationship between our body’s neurological and digestive systems. The figure was created using BioRender (https://www.biorender.com/) (accessed on 1 January 2024).

Probiotics, derived from the Greek word meaning “for life,” are living microorganisms that enhance the host’s health by improving microbial balance in the gut. They are primarily used to prevent or treat gastrointestinal issues through various mechanisms, such as the production of antimicrobial substances, modulation of the immune system, strengthening of the mucosal barrier, and competition with pathogenic bacteria for adhesion sites. Figure 2 presents a comprehensive view of the impacts psychobiotics have on neuroinflammation and neuropsychiatric disorders within the context of schizophrenia. The diagram delineates three core areas: Firstly, it highlights the factors contributing to the pathology, such as neuroinflammation and intestinal dysbiosis, emphasizing the role of IL-6 and TNF-α. Secondly, it illustrates the factors that can increase these inflammatory cytokines, including bacterial lipopolysaccharides (LPS). Thirdly, the diagram depicts the effects of inflammation on neurotransmitters like serotonin and dopamine. The central role of psychobiotics is to modulate this environment through mechanisms such as signaling via the vagus nerve, producing neuroactive metabolites, and inhibiting beta-amyloid fibrils. Probiotics and their ability to influence the formation and degradation of amyloid-beta (Aβ) fibrils represent an area of growing interest in the search for therapeutic strategies for neurodegenerative diseases, particularly Alzheimer’s Disease (AD). The formation of neuritic plaques composed of Aβ peptides is a central pathological feature of AD, and the accumulation of these peptides in the brain is a key factor in the disease’s pathogenesis. In this context, the capability of specific microbial enzymes, such as cysteine proteases and zinc metallopeptidases, to degrade Aβ peptides has been investigated. In vitro and in silico studies have demonstrated that these enzymes can cleave Aβ peptides at specific sites, suggesting a potential mechanism by which probiotics or their enzymes could influence the reduction of the Aβ peptide burden in the brain and, therefore, offer a therapeutic approach for AD. For example, it has been shown that cysteine protease can cleave the Aβ peptide between Lys16 and Cys17, while other enzymes have demonstrated the ability to degrade Aβ at different sites. From a therapeutic perspective, the identification and characterization of microbial enzymes that can efficiently degrade Aβ peptides open new avenues for drug design against AD [74,75]. This activity not only increases the level of neurotransmitters but also stabilizes the gut–brain axis and regulates enteroendocrine hormones, thereby offering a novel therapeutic approach for schizophrenia [8,9,13].

Given the global challenge of oral diseases like dental caries and periodontal disease, the limitations of traditional treatments like antibiotics, and psychiatric diseases like schizophrenia, there is a growing interest in alternative therapies [76].

The gut microbiota is increasingly recognized for its fundamental role in modulating physical and mental health [50]. The interaction between external and internal factors and the microbiota can affect the efficacy of pharmacological treatments, including antipsychotics used in managing psychiatric disorders like schizophrenia [77]. Below are some factors that affect the gut microbiota [77]:

○ Age: aging is associated with a reduction in the diversity of the intestinal microbiome, altering its configuration and potentially impacting overall health.
○ Diet and Nutrition: eating habits and periods of malnutrition or overnutrition are significant change agents for the gut microbiota.
○ Substance and Medication Use: The use of substances, including laxatives, antibiotics, and antipsychotics, has the potential to alter the composition of the microbiota. These changes can influence the bioavailability and efficacy of various medications.
○ Physical Exercise: Physical activity promotes the diversity of the microbiome, suggesting a positive link between exercise and intestinal health.
○ Geographic Location: the composition of the gut microbiota can vary significantly according to an individual’s geographic location, possibly due to differences in diet and environment.
○ Sampling Time: diurnal variability in sampling can reflect differences in microbial abundance and diversity, related to the food intake cycle.

Research on psychobiotics represents a significant advancement in understanding how the gut microbiota influences mental health. This field of study, which has evolved from early discoveries about the bidirectional communication between the brain and gut in the 19th and 20th centuries, experienced a major milestone with the coining of the term psychobiotics by Professor Ted Dinan in 2013. Unlike conventional probiotics, psychobiotics have the potential to positively affect mental health by influencing the production of neurotransmitters, SCFAs, and enteroendocrine hormones. This therapeutic impact has been observed in rodent studies, where supplementation with specific probiotics could reverse alterations in the microbiota and reduce symptoms of stress-induced leaky gut, as well as in human research that reported reductions in anxiety symptoms in individuals with depression following the administration of psychobiotics [13,14].

The popularity of and interest in psychobiotics have been on the rise, as evidenced by the growth in patent filings and the availability of products based on these compounds in the market. Psychobiotics have been shown to be effective in treating a wide range of neurological disorders, including stress, anxiety, insomnia, and neurodegenerative diseases like Alzheimer’s and Parkinson’s. The underlying mechanisms of their effects include immune activation, signaling through the vagus nerve, and the production of specific neuroactive metabolites. Additionally, psychobiotics offer neuroprotective functions, such as the inhibition of beta-amyloid fibril formation and antioxidant properties, highlighting the interconnection between the CNS and gut microbes [8,14].

The benefits of prebiotics and probiotics extend beyond the improvement of the gastrointestinal tract to mental health and metabolism. Prebiotics, as substrates selectively used by microorganisms that benefit health, and probiotics, living microorganisms that offer benefits when administered in adequate amounts, have shown potential to reduce anxiety, depressive behaviors, and improve cognition. The metabolites of probiotics, particularly SCFAs, play critical roles in colon health, the regulation of inflammation, and metabolism, in addition to influencing the permeability of the blood–brain barrier and the metabolism of tryptophan to serotonin [14]. Psychobiotics have been observed to stimulate the production of important hormones like cholecystokinin (CCK), peptide tyrosine tyrosine (PYY), and glucagon-like peptide-1 (GLP-1), which may have more potent effects than traditional probiotics in the body. This influence extends to the production of key neurotransmitters like dopamine, norepinephrine, GABA, serotonin, and acetylcholine, which are essential for the optimal functioning of the brain and CNS (CNS). The modulation of these neurotransmitters is facilitated through the established connection between the enteric nervous system (ENS) and the CNS via the vagus nerve, driven by the metabolism of indigestible fibers by various bacterial families [13,14,66].

The role of corticotropin-releasing hormone (CRH) in managing gastrointestinal function and its implication in stress-induced dysfunction, mucosal immune modulation, and inflammatory responses in the colon underscores the complexity of interactions between the nervous system and the gut microbiota. A serotonin imbalance in the gut has been linked to various diseases, and altered levels of neurotransmitters in germ-free (GF) mice demonstrate the profound connection between the microbiota and serotonergic signaling. Interestingly, certain strains of lactic acid bacteria have the capacity to synthesize nitric oxide (NO), a neurotransmitter crucial in various biological processes. Modulating the gut microbiota in adult mice can induce changes in behavioral patterns, suggesting that interventions affecting the microbiota may have significant implications in the treatment of disorders related to immune, hormonal, or neuronal mechanisms. This set of findings illustrates the importance of the gut microbiota not only in digestive health but also in regulating critical functions of the nervous system and in the potential mitigation of harmful effects [9,14].

The mechanisms of action of probiotics focus on protecting and strengthening the intestinal barrier function, modulating the immune system, influencing the host’s microbiota, modulating metabolic responses, and effects on the CNS. These mechanisms include the following [78]:

- Protection of Intestinal Barrier Function: Probiotics help maintain the integrity of the intestinal epithelium by promoting tight junction proteins, such as claudins, Zona occludin-1, and occludin, whose levels are significantly reduced in some disease conditions. Probiotics, like *Lactobacillus rhamnosus* GG, secrete effector molecules that stimulate the activation of ADAM17 and the release of HB-EGF, resulting in the transactivation of the Epidermal Growth Factor (EGF) receptor, prevention of apoptosis, and preservation of intestinal epithelial function.
- Stimulation of the Immune System: probiotics contribute to the maturation of the immune system, inducing the production of IL-10 in peripheral blood mononuclear cells, which facilitates the production of IgA antibodies at mucosal sites and enhances the immune response.
- Modulation of the CNS: some probiotics can produce neurotransmitters like GABA, influencing neuronal activity in the gastrointestinal tract, possibly affecting the CNS through vagus-nerve-mediated communication.
- Influence on the Host’s Microbiota: probiotics can modify the composition and function of the host’s gut microbiota by producing antimicrobial compounds that suppress or promote the growth of certain microorganisms in the gut, thus contributing to a healthy balance of the microbiota.
- Modulation of Metabolic Responses: probiotics can produce conjugated linoleic acid (CLA) and other compounds that regulate the expression of tight junction proteins and antioxidant enzymes, reducing oxidative stress in colonocytes and modulating inflammation.
- Cholesterol Reduction: some probiotics have the ability to produce bile salt hydrolase, which hydrolyzes conjugated bile salts; this releases less-soluble primary bile acids that are excreted rather than reabsorbed, contributing to the reduction in blood cholesterol levels.

Neurons and enteroendocrine cells can synthesize serotonin from the precursor amino acid L-tryptophan, whereas platelets depend on the uptake of serotonin for their reserves. The synthesis of serotonin initially involves the conversion of L-tryptophan to 5-hydroxytryptophan by the enzyme L-tryptophan hydroxylase (TPH), with this step being the rate-limiting step for serotonin synthesis. The identification of two isoforms of this enzyme, TPH1 and TPH2, suggests the possibility of finding drug inhibitors specific to each isoform in the future. Serotonin is also primarily metabolized by the enzyme monoamine oxidase (MAO), with two subtypes, MAO-A and MAO-B, present in the brain and peripheral tissues. The regular use of psychobiotic microorganisms plays a direct role in the immune balance of the host [79].

These mechanisms underscore the complexity of the interactions between probiotics and the host, highlighting the therapeutic potential of probiotics in the prevention and treatment of various diseases [78].

Clinical research has demonstrated that psychobiotics can offer significant benefits in the treatment of neuropsychiatric disorders, including schizophrenia. Studies have revealed differences in the composition of the gut microbiome between patients with schizophrenia and healthy controls, showing the influence of certain bacteria on the manifestation of negative and depressive symptoms. Additionally, combined supplementation of vitamin D and probiotics has resulted in notable improvements in schizophrenic patients, suggesting the potential of probiotics to improve both disease symptoms and associated intestinal discomforts [8,13,14].

A pioneering trial that used a combination of *Lactobacillus rhamnosus* GG and *Bifidobacterium animalis* subsp. *Lactis* Bb12 found no significant differences in the severity of psychiatric symptoms compared to the placebo. However, this probiotic compound demonstrated a notable reduction in intestinal difficulties and significant alterations in the levels of various serum proteins, suggesting a positive impact on gastrointestinal well-being and potential systemic effects on the body [80].

*Bacillus coagulans* MTCC 5856 has been shown to be effective in managing symptoms of irritable bowel syndrome (IBS) and major depression, while Bifidobacterium longum (strains 1714 and NCC3001) has been associated with stress reduction and memory improvement. *Clostridium butyricum* MIYAIRI 588 has proven effective in combination with antidepressants in treating resistant depression, and *Lactobacillus casei Shirota* has been shown to decrease anxiety and improve sleep quality. Additionally, combinations of strains, such as *Lactobacillus helveticus* R0052 and *Bifidobacterium longum* R0175, have resulted in a significant decrease in depression, highlighting the importance of synergy between different probiotic strains [9]. Key outcomes include the reduction of depression and anxiety through the consumption of specific probiotics, such as *Lactobacillus helveticus* R0052 and *Bifidobacterium longum* R0175, which have shown significant benefits in psychological behavior and biomarkers in humans. Other strains, like Lactobacillus casei Shirota, have been shown to reduce cortisol levels and improve mood states in stressful situations [81].

However, the results of clinical trials with probiotics in patients with schizophrenia have been mixed, with no significant differences in symptom severity between the probiotic and placebo groups. Despite this, immunomodulatory effects have been noted in patients with treatment-resistant schizophrenia, including cytokine modulation and improvements in gastrointestinal function. This finding highlights the value of probiotics as a complement to atypical antipsychotics. Additionally, the association between CD14 seropositivity and antibodies against *C. albicans* and gluten with an increased risk of schizophrenia underscores the importance of the interaction between the immune system and the gut microbiota in the pathogenesis of the disease [82]. Although studies indicate a promising path towards managing mental illnesses through modulation of the gut microbiota, the need for further research to confirm the efficacy of probiotics and determine the most beneficial specific strains is evident [82]. The need for rigorous surveillance regarding the safety and regulation of probiotic use is highlighted due to their variability in effects and strain specificity. In conclusion, manipulating the intestinal microbial balance opens new avenues for the treatment of psychiatric disorders, emphasizing the relevance of the gut microbiome in overall mental health and the specific management of schizophrenia [8,13,14].

An initial trial in schizophrenia with an additional probiotic compound (*Lactobacillus rhamnosus* GG and *Bifidobacterium animalis* subsp. *Lactis* Bb12) showed no significant differences in the severity of psychiatric symptoms between the probiotic group and placebo. However, patients who received the probiotic compound were less likely to develop severe intestinal problems, indicating a positive effect on the gastrointestinal tract [53]. Additionally, probiotic supplementation significantly altered the levels of various serum proteins and reduced antibody levels against Candida albicans and gastrointestinal symptoms in men from the trial. The article also notes that approximately 50% of patients with schizophrenia experience constipation, which can be a serious issue, especially for those treated with clozapine [53].

Probiotics and psychobiotics, including specific strains of Lactobacillus and Bifidobacterium, have shown promising effects in reducing depressive and anxiety symptoms in both animal models and human trials. These effects are attributed to various mechanisms, such as neurotransmitter modulation (GABA, serotonin), immune function enhancement, the reduction of inflammatory markers, and influences on the gut–brain axis. Key outcomes include the reduction of depression and anxiety through the consumption of specific probiotics, such as *Lactobacillus helveticus* R0052 and *Bifidobacterium longum* R0175, which have demonstrated significant benefits in psychological behavior and biomarkers in humans. Other strains, like *Lactobacillus casei Shirota*, have been shown to reduce cortisol levels and improve mood states in stressful situations [81].

Table 2 of the study provides a detailed overview of specific psychobiotics with therapeutic potential in the treatment of schizophrenia, highlighting their effects and potential mechanisms of action. Among them, *Lactobacillus rhamnosus* (JB-1) shows a notable ability to reduce stress-induced corticosterone levels and depressive behaviors by downregulating HPA axis activity and altering GABA receptor expression. *Mycobacterium vaccae* is associated with reduced anxiety in maze learning tasks, although its specific mechanism has not been specified. *Bifidobacteria infantis* stands out for increasing tryptophan and serotonin levels and decreasing pro-inflammatory cytokines, exerting immunomodulatory effects and modulating tryptophan metabolism. *Lactobacillus helveticus* NS8 shows anti-inflammatory effects, reduces post-restriction anxiety, improves memory, and decreases corticosterone and ACTH levels, with an increase in BDNF mRNA in the hippocampus. Additional research on *Lactobacillus rhamnosus* JB-1 revealed increases in the concentrations of glutamate, GABA, and tNAA, indicating changes in neural metabolism and neurotransmitter concentrations modulation, particularly in the excitatory and inhibitory balance. Finally, it is observed that General Probiotics reduce gastrointestinal inflammation, immune activation, and modulate physiological variables, including inflammatory markers, through anti-inflammatory properties, stimulation of the vagus nerve, and cytokine modulation [5,52,81].

Growing evidence suggests that the composition and diversity of the gut microbiome can significantly influence mental and physical health, opening new avenues for understanding and treating complex disorders. In this series of studies, researchers from various fields have explored how differences in the gut microbiome between patients with schizophrenia and healthy controls could be related to symptom severity, brain structure and function, cognition, and metabolism. These findings highlight the potential of the gut microbiome as a therapeutic target and underline the importance of multidisciplinary research in unraveling the complex interactions between our internal microbial environment and mental health [86,87].

Nocera and Nasrallah, 2022 [86], conducted a systematic review to explore the relationship between the gut microbiome and schizophrenia, finding notable differences in the composition and abundance of certain bacterial families and genera between patients with a first psychotic episode (FEP) and healthy controls (HCs). Specifically, bacteria such as *Lachnospiraceae, Bacteroides* spp., and *Lactobacillus* spp. were correlated with increased severity of schizophrenic symptoms, including negative and positive symptoms, as well as a decrease in overall functionality. Most studies analyzed did not observe significant changes in the alpha diversity of the microbiome between patients and controls, consistent with previous research. Despite variations in the associations of specific bacteria with clinical features of schizophrenia and the absence of a clear pattern in diversity measures, these findings suggest that different characteristics of schizophrenia may be reflected in the gut microbiome. This link between the microbiome and the severity of negative symptoms, particularly challenging in treatment and contributing to functional impairment in schizophrenia, offers a promising perspective for identifying biomarkers and developing new microbiome-based intervention strategies [86].

Xu et al., 2019 [88], studied 168 participants, divided between patients with schizophrenia and healthy controls, to explore the composition of their gut microbiota using metagenomic sequencing and 16S rRNA gene sequencing, revealing significant differences in microbial diversity and composition between both groups. Specific patterns of dysbiosis in patients were identified, including a decrease in microbial richness and alterations in specific taxonomies and metabolic functions related to glutamate metabolism and mucosal immunity. Elevated levels of intestinal IgA and increased activity of glutamate synthase (GOGAT) in patients point to an interaction between altered microbiota and intestinal immunity.

A study by Li et al., 2021 [89], is pioneering in investigating and finding a significant correlation between the gut microbiome and brain structure and function in patients with schizophrenia. It revealed notable differences in microbiome composition between patients and healthy controls, as well as positive associations between the alpha diversity of the microbiome and gray matter volume indices and regional homogeneity in several key brain regions. The analysis also highlighted how alcohol and tobacco consumption affects microbiome composition, increasing intestinal permeability and endotoxin translocation, which could influence schizophrenia pathology. Moreover, the study identified that the decrease of short-chain-fatty-acid-producing bacteria, such as *Roseburia* spp., may have negative effects on insulin sensitivity and brain serotonin, suggesting a link between the microbiome and alterations in brain structure and function observed in schizophrenia. This work underscores the complex interaction between the gut microbiome and schizophrenia, opening new avenues for understanding and treating the disease [89].

The study by Yang et al., 2022 [90], offers a bibliometric analysis of the relationship between schizophrenia and the gut microbiota, highlighting the growing research since 2014 with contributions from 873 authors from 355 organizations in 40 countries. It identifies Timothy Dinan, John F. Cryan, and Emily Severance as key figures and institutions such as Johns Hopkins University, University College Cork, and the University of Toronto as leaders in this field. It reveals that the United States, China, and Canada are the main contributors and points to journals like *Schizophrenia Research* and *Frontiers in Psychiatry* among the most productive. The study also outlined four main research directions based on keyword co-occurrence, ranging from gut microbiota and bipolar disorder to schizophrenia and its treatment implications, the gut–brain axis in autism and depression, and the role of inflammation in brain health [90].

A study by Thirion et al., 2022 [87], examines differences in the gut microbiome of patients with schizophrenia, especially those with an increase in waist circumference, compared to healthy controls and people with metabolic syndrome. Through metagenome sequencing of fecal samples, it was discovered that the microbiome of individuals with schizophrenia has a unique composition and richness, highlighting an enrichment of species such as *Flavonifractor plautii* and a reduction in *Faecalibacterium prausnitzii*. Significantly, the study reveals that 11% of cognitive variability in patients with schizophrenia can be explained by the functional potential of the microbiome, finding a positive correlation between bacterial biosynthesis of tyrosine, a precursor of dopamine, and cognition. These results suggest marked differences in microbiome composition between schizophrenic patients and control groups, implying that the gut microbiome could be an intervention point to mitigate cognitive dysfunction associated with schizophrenia. This discovery emphasizes the relevance of the gut–brain–microbiome axis in schizophrenia and promotes future research to explore microbiome manipulations that could influence cognition through tyrosine biosynthesis [87].

A study by Shi et al., 2022 [91], investigates the relationship between schizophrenia and alterations in the intestinal microflora and imbalances in intestinal metabolites, using 16S ribosomal RNA sequencing and metabolic profiles to analyze samples from 44 healthy controls, 41 patients in the acute phase, and 39 in remission. Twenty distinct dominant intestinal microfloras were identified among the groups, including changes at the levels of orders, families, genera, and species. One hundred forty-five unusual microbial metabolites were found between the acute and remission groups, involved in human metabolism and disease processes according to the *Kyoto Encyclopedia of Genes and Genomes*. The results showed significant differences in intestinal microbial composition and fecal metabolites between acute patients, those in remission, and healthy controls, with a correlation between these changes and the severity of schizophrenic symptoms. Specifically, a distinct beta diversity was observed among the groups, indicating a potential interactive influence of the gut microbiota and its metabolites on the pathophysiology of schizophrenia. This study suggests that the gut microbiota and its metabolites could influence brain function and behavior through the gut–brain axis, predisposing individuals to the development of schizophrenia [91].

A study by Liu et al., 2021 [52], explores how the gastrointestinal microbiome affects the gut–brain axis and its relationship with psychiatric disorders such as schizophrenia. Using models in mice and humans, it was found that an altered microbiome can influence abnormal behaviors and be associated with schizophrenia, highlighting the importance of the gut–brain axis in its pathological mechanisms. Despite variability in results, there is a consensus that the microbiome in schizophrenic patients is distinct from that of healthy individuals. The study notes the need for further research to determine the microbial specificities related to schizophrenia and the factors affecting the microbiome [52].

A study by Nita et al., 2022 [92], offers a comprehensive review of therapeutic approaches aimed at modulating the gastrointestinal microflora in patients with schizophrenia, highlighting the potential of supplements derived from microorganisms and specific protocols to restore eubiosis in the host. The research was based on a search of the relevant literature in four databases, identifying twenty-two eligible cases focused exclusively on experiences with human patients. It was found that the administration of specific strains of lactic acid bacteria, such as *Lactobacillus* spp. and *Bifidobacterium* spp. or combinations of these with vitamin D and selenium, helps maintain the integrity of the intestinal flora. These interventions prevent adverse effects associated with the use of antipsychotics, such as inflammation and weight gain related to olanzapine, and other metabolic dysfunctions. However, the study also notes that several antipsychotics have a potent effect on the intestinal flora, altering the balance between opportunistic pathogens and beneficial bacteria, suggesting the need for further research in this area.

Minervini et al., 2023 [93], conducted a systematic review and meta-analysis of the use of probiotics in the treatment of oral mucositis induced by radiotherapy, showing beneficial effects in reducing the severity of mucositis in patients with head and neck cancer.

McGuinness et al., 2022 [94], examined how the gut microbiota, considered a type of metabolic machinery that influences many aspects of physiology, is associated with major psychiatric disorders such as Major Depressive Disorder (MDD), bipolar disorder (BD), and schizophrenia. Through a systematic review following PRISMA guidelines and analyzing 44 studies that included a total of 2510 psychiatric cases and 2407 controls, the study aimed to identify differences in the composition of the gut microbiota between individuals with these psychiatric disorders and healthy control, as well as to explore possible similarities in the microbial signatures associated with these disorders. The findings did not show strong evidence of differences in the number or distribution (α-diversity) of bacteria in people with mental disorders compared to controls. However, there was consistency in reporting differences in the overall community composition (β-diversity) between individuals with mental disorders and controls. Specific bacterial taxa commonly associated with mental disorders were identified, including lower levels of short-chain-fatty-acid-producing bacterial genera (e.g., butyrate), higher levels of lactic-acid-producing bacteria, and higher levels of bacteria associated with the metabolism of glutamate and GABA. The review also highlights substantial heterogeneity among studies in terms of methodologies and findings [94].

A study by Liu et al. (2021) [52], on the diversity of study designs and findings regarding differences in taxon abundance in schizophrenia across various studies highlights the complex relationship between the gut and oral microbiomes and schizophrenia. The key points of the mentioned studies are summarized as follows:

He et al. (2018) [95]: This study focused on the gut microbiome of individuals at high and ultra-high risk for schizophrenia, along with healthy controls. Using 16S rRNA gene sequencing, no significant differences were found in alpha diversity, but there was an increased relative abundance of specific taxa such as *Lactobacillus* spp. and *Prevotella* spp. in schizophrenia patients.

Castro-Nallar et al. (2015) y Yolken et al. (2020) [96]: these studies explored the oral microbiome in schizophrenia patients, finding a lower species richness and altered beta diversity, with an increase in the abundance of genera such as Bifidobacterium and Streptococcus and a decrease in the abundance of Prevotella and *Neisseria subflava* in schizophrenia patients.

Zhang et al. (2020) y Zhu et al. (2020) [97]: These studies investigated the gut microbiome in first-episode schizophrenia patients who had not received antipsychotic treatment. The findings indicated no significant changes in alpha diversity but significant differences in beta diversity, with an increase in the abundance of Proteobacteria and other specific taxa and a decrease in the abundance of *Lachnospiraceae* in the schizophrenia group.

Yuan et al. (2018) [98]: they studied the impact of risperidone treatment on the gut microbiome of first-episode schizophrenia patients, observing changes in the abundance of *Bifidobacterium* spp., *Escherichia coli*, *Lactobacillus* spp., and *Clostridium coccoides.*

Pełka-Wysiecka et al. (2019) [99], Shen et al. (2018) [100], Nguyen et al. (2019) [101], Ma et al. (2020) [102], and Xu et al. (2020) [103]: These studies further explored the gut microbiome in schizophrenia patients, reporting various alterations in microbial diversity and taxon abundance, with some studies noting a lower alpha diversity and changes in the Firmicutes/Bacteroidetes ratio in patients treated with antipsychotics.

A study by Zagórska et al., 2020 [104], investigates the impact of psychobiotics, which are live microorganisms that, when ingested in adequate amounts, can offer benefits for mental health. This systematic analysis and meta-analysis of 23 clinical studies with 2726 participants shows that probiotic administration can significantly improve the symptoms of depression but did not have a significant effect on anxiety, stress, or schizophrenia. The most used probiotics were from the genera Lactobacillus and Bifidobacterium, and the studies varied in terms of strains used, duration of the intervention, and populations studied, which introduced heterogeneity in the results. The specific mix and quantity of probiotic strains, along with the form of administration (such as capsules, powders, or fermented foods), may influence the effectiveness observed in different studies.

The diverse studies, ranging from systematic reviews to meta-analyses and bibliometric analyses, collectively reveal notable differences in the microbial composition and diversity between individuals with schizophrenia and healthy controls. These differences are not only evident in the abundance of specific bacterial families and genera but also in the functional potential of the microbiome, which appears to influence the pathophysiology and symptomatology of schizophrenia, including cognitive functions and metabolic processes. The findings suggest a promising avenue for the development of new treatment strategies and interventions targeting the gut microbiome. By manipulating microbial composition or metabolic output, it may be possible to mitigate some of the challenging symptoms associated with schizophrenia, particularly those that are resistant to traditional treatments.

### 3.7. Revolutionizing Gut Health: The Promise of Artificial Intelligence, Nanotechnology, and Synthetic Biology in Psychobiotics

At the forefront of contemporary science, AI and synthetic biology (SB) are emerging as revolutionary tools in the research on the gut microbiome, offering new dimensions for understanding and manipulating this complex ecosystem with significant implications for human health. The application of machine learning algorithms to the study of the microbiome has enabled in-depth analysis of complex biological interactions, facilitating the identification of hidden patterns, disease prediction, and the discovery of potential biomarkers. This integration of advanced technology promises to transform not only our understanding of chronic, inflammatory, and neurological diseases but also the way they are therapeutically approached [20,105,106,107].

The use of deep learning techniques and neural networks in the analysis of multi-omics data has been particularly impactful, allowing for the precise prediction of diseases and the personalization of therapies and diets based on individual microbiotic profiles. AI has been applied to discover microbial markers that serve as indicators of optimal gut health or the presence of specific disorders, revealing how probiotic supplementation could favorably alter the microbiome composition [21,106,108,109].

On the other hand, SB combines principles of biology and engineering to design biological entities with new functionalities, such as genetically modified probiotics and bacteriophages, opening pathways towards the therapeutic manipulation of the microbiome. CRISPR-Cas genome editing has revolutionized the ability to precisely modify microbial genomes, enhancing the development of probiotics with improved therapeutic capabilities. Similarly, the construction of genetic circuits and communication systems allows controlled interactions between modified and native gut microbes, offering novel strategies to beneficially modify the microbiome composition [105,110].

However, these advances are not without challenges, including the complexity of generalizing AI models across diverse populations and the safety and stability of modified microbes within the complex intestinal ecosystem. Effective integration of multi-omics data and the development of microbes with enhanced functionalities require addressing these difficulties to fully leverage their potential [81,105].

The future of analyzing and manipulating the gut microbiome through AI and SB is promising, with the potential to revolutionize our understanding and management of multiple health conditions. As we move forward, the development of more robust and generalizable methods, as well as meticulous scrutiny and regulatory authority approval for the clinical use of these innovative technologies, is crucial. The synergy between AI, SB, and microbiome research opens a new horizon in personalized medicine, heralding an era where gut health and, by extension, general health can be optimized in a precise and personalized manner [19,105].

A study by Westfall et al., 2021 [21], introduces an artificial model of the human gastrointestinal tract, named ABIOME, to optimize probiotic therapies using machine learning. This dynamic modular model allows real-time monitoring of gastrointestinal conditions and supports complex cultures similar to the human microbiota. Using ABIOME, optimized probiotic formulations were designed through multivariate adaptive regression spline (MARS) analysis, identifying combinations that produce bioactive metabolites with potential therapeutic benefits. This innovative approach promises significant advances in the creation of probiotics specific to therapeutic applications based on their metabolic activity.

A study by McCoubrey et al., 2022 [19], applied automated active learning to predict how excipients affect the growth of the probiotic *Lactobacillus paracasei*. Starting with a dataset of six bacteria–excipient interactions, they successfully predicted the effects of 111 untested excipients with an average certainty of 67.70%. Experimental validation confirmed that three out of four excipient–probiotic interactions were correctly predicted. This approach demonstrates that excipients have significant effects on the proliferation of probiotics and underscores the importance of thoughtful design for the oral delivery of precision probiotics, optimizing intestinal microbial colonization and subsequent therapeutic benefits. This work pioneers the application of automated active learning in microbiome science and highlights its utility of working with small datasets in pharmaceutical sciences.

A study by Forth et al., 2023 [111], significantly broadens our understanding of the role of probiotics as an adjunctive treatment in psychiatric disorders. The systematic review addresses how probiotics and synbiotics can enhance the efficacy of frontline treatments for disorders such as Major Depressive Disorder (MDD) and Generalized Anxiety Disorder (GAD), based on evidence from eight studies that met specific eligibility criteria. The findings suggest that adding probiotics to standard treatment may be more effective in improving psychiatric symptoms than standard treatment alone or the use of a placebo, especially in cases of MDD and GAD. For patients with schizophrenia, although adjunctive treatment with probiotics did not show significant differences in clinical outcomes, it did improve the tolerability of first-line antipsychotics, suggesting a potential benefit in managing the side effects of antipsychotic treatment. The review also highlights the importance of the MGB axis in the pathogenesis and treatment of psychiatric disorders, suggesting that probiotics may offer a complementary therapeutic approach by modulating this axis. The conclusion of the study proposes that supplementation with probiotics, alongside Selective Serotonin Reuptake Inhibitors (SSRIs), could significantly enhance treatments for MDD and GAD compared to SSRIs alone. Although benefits in the tolerability of treatments for schizophrenia were observed, there were no significant improvements in clinical outcomes. This study emphasizes the need for further research to more deeply explore the impact of probiotics on the treatment of psychiatric disorders and their mechanisms of action, highlighting the potential of probiotics as a promising adjunctive treatment in psychiatry.

A study by Laterza et al., 2023 [106], explores the potential of AI in identifying and applying probiotics to improve gastrointestinal health and, by extension, overall well-being. Using AI models, the study delves into the complex ecosystem of the intestinal microbiome to predict how different probiotic strains might influence various health conditions. The findings underscore the ability of AI to analyze large sets of microbiological data, allowing for the customization of probiotic treatment by identifying specific combinations of strains that are most beneficial for particular conditions. The research highlights the use of advanced algorithms to discover microbial markers that could serve as indicators of optimal intestinal health or the presence of specific disorders.

The array of studies presented underlines the transformative potential of integrating AI and synthetic biology (SB) in the realm of probiotics and gut microbiome management. These innovative methodologies and analytical approaches herald a new phase in personalized healthcare, where probiotic therapies are not just generalized but tailored to individual metabolic profiles and health conditions. These advancements signify a leap towards a future where precision in probiotic application enhances not only gastrointestinal health but also contributes significantly to the holistic well-being of individuals.

### 3.8. Advancements and Challenges in the Application of Nanoprobiotics and Nanomedicine

The innovation of nanoprobiotics, which combines probiotics with nanotechnology, offers advanced solutions for administration and effectiveness in the oral cavity, through nanoencapsulation techniques that protect probiotics and ensure targeted delivery to oral tissues. These advancements promise to improve treatments against dental caries and periodontal diseases by inhibiting the growth of pathogens such as Streptococcus mutans and *Porphyromonas gingivalis*. However, the application of nanoprobiotics in oral health requires further research to optimize the formulation, assess safety, and confirm efficacy through expanded clinical trials, opening a promising path towards alternative therapies in oral health and the expansion of probiotics use in medicine and food [76].

Brain diseases and gastrointestinal and oral conditions face promising innovations through nanotechnology and probiotic microencapsulation, respectively. Nanotechnology, with its nanocarriers and nanoparticles, offers a way to overcome the blood–brain barrier and improve drug delivery for treating diseases such as Alzheimer’s and multiple sclerosis. In parallel, microencapsulation protects probiotics, enhancing their viability and delivery in the gastrointestinal tract, with potential benefits for colon health and disease prevention [112,113]. However, both approaches face challenges, including the potential toxicity of nanomaterials and the need for further research on the safety and efficacy of these technologies. Despite these obstacles, nanotechnology and microencapsulation represent significant advancements in the treatment of brain diseases and in improving gastrointestinal and oral health, marking the path towards future innovations in the medical field [16,17].

A study by Pandey et al., 2024 [114], delves into the growing interest in probiotics and their potential as medicinal adjuvants, highlighting their role in improving digestion, nutrition, metabolism, and strengthening the immune system, as well as in regulating various body functions. It emphasizes the need to ensure the effective delivery of probiotics to the intestine through advanced technologies like nanotechnology, which allows the encapsulation of probiotics to increase their viability and bioavailability. This study underlines the importance of specific criteria for the identification of probiotic strains, in accordance with FAO/WHO guidelines, and explores the use of lactobacilli as potential probiotics, given their classification as GRAS and QPS by regulatory bodies. Through an exhaustive analysis and following PRISMA guidelines, studies on the encapsulation of probiotics are reviewed, demonstrating the efficacy of nanotechnology in enhancing the functionality of probiotics, with a particular focus on *Lactobacillus* spp. The work concludes by highlighting the value of the probiotic food market and the need for effective regulations to ensure the safety and efficacy of these products.

Nanotechnology has revolutionized the encapsulation and delivery of probiotics, using a variety of nanostructured materials to improve the viability and effectiveness of probiotics [115]. Among these materials are nanocellulose, with its crystals and nanofibers offering biocompatibility and low toxicity; magnesium oxide nanoparticles, noted for their high surface area and mechanical strength; chitosan nanoparticles, with cationic properties and biocompatibility; Eudragit S100 nanoparticles, which vary their solubility with pH; and starch nanoparticles, used in biomedical applications for their abundance in nature [16,116].

Furthermore, nanoparticles for drug delivery in the treatment of CNS disorders include liposomes, which can encapsulate a wide range of therapies; polymeric nanoparticles, offering protection against enzymatic degradation; solid lipid nanoparticles, with physical stability and low toxicity; micelles, to improve the solubility of lipophilic drugs; nanoemulsions, which enhance the bioavailability of poorly soluble drugs; dendrimers, for drug conjugation; and quantum dots, useful for imaging and diagnosing CNS disorders [117,118].

Microencapsulation of probiotics employs techniques such as spray drying, emulsification, and extrusion to protect them from adverse environmental factors and enhance their delivery in the gastrointestinal tract. Electrospinning technology produces continuous nanofibers for various applications, including the encapsulation of probiotics, improving their tolerance to hostile environments. The encapsulation materials range from polysaccharides like alginate and chitosan, which offer protection in acidic environments and enhance the viability and targeting of probiotics, to proteins and lipids that exhibit amphiphilic properties and efficient nanocapsule formation [80,119].

Challenges in probiotic encapsulation include the need for materials that allow the release of probiotic cells at the desired site through environmental stimuli, and the development of new and innovative microcapsules for better protection of probiotics (see Table 3). The quality assessment of probiotic encapsulation is based on criteria such as particle size, viability, and encapsulation efficiency, which are critical for determining the effectiveness of encapsulated probiotics [16,18,80,114].

Nanotechnology, particularly through the advent of DNA-based nanodevices, is poised to redefine therapeutic approaches, heralding a new era in the precise delivery of probiotics and psychobiotics [120]. These devices harness the programmable nature of DNA to create nanostructures that can interact with biological systems in precise and controllable ways. DNA origami, a technique that folds DNA into predefined two- and three-dimensional shapes, serves as the foundation for constructing these nanoscale devices [121,122]. By designing specific DNA sequences, researchers can create structures that respond to environmental stimuli, enabling the controlled release of therapeutic agents at targeted sites within the body. A key strength of DNA nanodevices lies in their potential to serve as innovative carriers that safeguard and convey probiotics or psychobiotics to specific areas within the gastrointestinal tract. This envisioned targeted delivery mechanism is poised to significantly boost the resilience of probiotics as they navigate through the stomach’s acidic conditions, ensuring that these beneficial microorganisms are liberated precisely where they can deliver their optimum health benefits [120]. Furthermore, DNA-based carriers can be designed to respond to specific biological signals, releasing their payload only in the presence of certain triggers, such as the pH levels typical of different parts of the intestine or specific biomarkers associated with disease states. The application of DNA nanotechnology in drug delivery extends beyond probiotics. By incorporating functional elements such as aptamers or enzymes, DNA nanodevices can be engineered to target cancer cells, release drugs in response to enzymatic activity, or modulate immune responses [120,123]. These capabilities open new avenues for creating more effective and personalized therapies for a range of conditions, including neurodegenerative diseases, where the gut–brain axis plays a crucial role.

**Table 3:** Nanoparticles and nanostructured materials for probiotic encapsulation.

Material Type	Description	Applications	Advantages	Disadvantages
Nanocellulose [16,18]	Available in CNC and CNF forms. Known for its low toxicity, biocompatibility, and adjustable surface properties. It has been shown to improve the properties of probiotic delivery systems when used as an encapsulating material.	Probiotic Encapsulation	Biocompatible and eco-friendly; provides mechanical strength and adjustable surface properties for better encapsulation.	Limited protection against extreme pH and enzymes.
Magnesium Oxide Nanoparticles (MgO NPs) [16,18]	Attracted attention due to its high surface area, non-toxicity, mechanical resistance, thermal stability, and low cost. Used for microencapsulation of probiotics, showing an improvement in probiotic viability in acidic environments.	Microencapsulation of Probiotics	Enhances probiotic viability in acidic environments; offers mechanical resistance and thermal stability.	Potential aggregation in biological media; requires careful surface modification.
Chitosan Nanoparticles (CSNPs) [16,18,114]	Derived from the alkaline deacetylation of chitin; chitosan is a natural polysaccharide with cationic properties, biocompatibility, non-toxicity, and low cost. Chitosan nanoparticles have shown promise for the encapsulation of probiotic cells, protecting them in the GI tract and improving their mucoadhesive properties.	Encapsulation and Protection in GI Tract	Biocompatible; non-toxic; enhances mucoadhesive properties, protecting probiotics in the GI tract.	Limited solubility in water and some solvents; potential deacetylation challenges.
Eudragit S100 Nanoparticles [16,18,114]	A synthetic anionic polymer derived from methacrylic acid and methyl methacrylate ester. Its solubility depends on pH, being insoluble in strongly acidic solutions and slightly soluble in regions of the digestive tract with neutral to weakly alkaline pH. Used to improve viability of probiotic bacteria.	Enhancing Probiotic Viability	Effective at protecting probiotics in acidic GI environments; pH-responsive solubility for targeted release.	Requires careful formulation to achieve desired solubility and release profiles.
Starch Nanoparticles [18,80]	One of the most abundant biopolymers in nature; produced by many plants and crops. Starch nanoparticles and nanocrystals have been used for biomedical applications, especially in drug delivery. Although, not the best candidate for probiotic microencapsulation due to potential immediate release in hostile environments.	Potential for Probiotic Encapsulation	Natural and biodegradable; potentially low cost.	Possible immediate release in hostile environments, modifications necessary for stability.
Liposomes [124]	Spherical vesicles composed of one or more lipid layers surrounding an aqueous core. Morphologically similar to cell membranes, they can encapsulate hydrophilic drugs in their aqueous core and lipophilic drugs in the lipid bilayer, making them versatile for the delivery of a wide range of therapies.	Wide Range of Therapy Delivery	Can encapsulate both hydrophilic and lipophilic compounds; biocompatible and versatile for various therapies.	Stability issues in the GI tract; potential for leakage or fusion with other lipids.
Polymeric Nanoparticles (PNPs) [124]	Colloidal mixtures of biocompatible and biodegradable polymers forming a dense matrix; capable of encapsulating lipophilic drugs within its structure. These nanoparticles offer steric stabilization, protection from enzymatic degradation, and controlled drug release.	Drug Delivery and Stabilization	Protection from enzymatic degradation; controlled release; steric stabilization.	Potential for immune response; complexity in manufacturing.
Solid Lipid Nanoparticles (SLNs) [124]	Colloidal dispersions of lipids that solidify at room or body temperature. They offer physical stability, drug protection, and low toxicity. Capable of encapsulating both hydrophilic and lipophilic drugs.	Drug Encapsulation	Physical stability; drug protection; low toxicity.	Limited drug loading capacity; potential for drug expulsion during storage.
Micelles [125]	Composed of amphiphilic molecules (having a hydrophilic and a hydrophobic part), micelles form core–shell structures where the hydrophobic core can encapsulate lipophilic drugs, improving their solubility and bioavailability.	Enhancing Solubility and Bioavailability	Improves solubility and bioavailability of lipophilic drugs; simple to prepare.	Critical micelle concentration dependent stability; potential dilution issues in vivo.
Nanoemulsions (NE) [124]	Colloidal systems containing oil, water, and surfactants; capable of improving the solubility of water-insoluble drugs and offering controlled drug release.	Solubility Improvement and Controlled Release	Enhances solubility of water-insoluble drugs; controlled release capabilities.	Physical stability over time can be challenging, requiring surfactants for stabilization.
Dendrimers [124]	Highly branched polymeric structures providing a platform for drug conjugation, aimed at improving solubility, stability, and efficacy of drug delivery across the blood–brain barrier.	Drug Delivery Efficiency	High drug loading capacity; targeted delivery potential; modifiable surface for functionalization.	Complexity in synthesis; potential toxicity depending on composition and dose.
DNA-based Nanodevices [120,122,123]	Utilizing the precision of DNA to form nanoscale structures, these devices are tailored for specific interactions within biological systems. They are especially promising for the accurate placement of probiotics or psychobiotics within the gastrointestinal tract.	Targeted Delivery of Probiotics and Psychobiotics	Precise control over delivery location; capable of protecting cargo through harsh conditions; programmable release triggered by environmental factors.	Complexity in design and synthesis; potential for unanticipated interactions with the body’s biochemistry.
Quantum Dots (QDs) [124,126]	Nanocrystalline semiconductors offering unique electronic and optical properties, such as high emission and photostability; useful for imaging and diagnosis of CNS disorders, as well as drug delivery.	Imaging, Diagnosis, and Drug Delivery	High photostability and emission for imaging; potential for targeted drug delivery.	Toxicity concerns, especially with heavy-metal-containing QDs; stability in biological environments

## 4. Conclusions

These new research findings on psychobiotics open up new perspectives on the interaction between gut microbiota and schizophrenia, suggesting a promising path toward innovative therapies. They highlight the ability of psychobiotics to positively influence the treatment of schizophrenia, underscoring the importance of dysbiosis in the pathogenesis of this complex disorder. The integration of advances in AI and nanotechnology emerges as a fundamental pillar for the personalization and optimization of these therapeutic approaches, offering a multidisciplinary strategy to address the complexities of the disease. Moreover, the application of AI and nanotechnology not only facilitates the tailoring of therapeutic interventions to individual patient profiles but also enhances the precision with which these treatments target the underlying biological mechanisms of schizophrenia. AI algorithms can analyze vast datasets from gut microbiome analyses to predict patient responses to specific psychobiotics, thereby streamlining the selection process for identifying the most effective treatment regimens. Concurrently, nanotechnology enables the engineering of delivery systems that can bypass the harsh gastrointestinal environment, ensuring that therapeutic agents reach their intended site of action intact. This symbiosis between cutting-edge technology and biomedical science paves the way for a future where treatments are not only more effective but also significantly reduce the burden of side effects, heralding a new era in the personalized and nuanced management of psychiatric disorders.

## Figures and Tables

**Table 1:** Factors affecting microbiota.

Factor	Impact on Gut Microbiota
Mode of Delivery [50,57,58]	Influences the initial colonization of a newborn’s GI tract. Vaginal delivery (VD) leads to a microbiota similar to maternal vaginal microbiota with a dominance of *Lactobacillus* spp. Cesarean section (CS) results in decreased diversity and an imbalance, with infants showing higher abundance of hospital pathogens and lower abundance of *Bifidobacteria* spp., *Bacteroides* spp., *Staphylococcus* spp., *Corynebacterium* spp., and *Propionibacterium* spp.
Probiotics [50]	Regulates the immune system, supports gut barrier integrity, and has been shown to alleviate symptoms of depression, anxiety, and stress. Specific strains like *Lactobacillus helveticus* R0052 and *B. longum* R0175 have shown benefits in mental health.
Stress [50,59]	Stress can decrease the number of beneficial species like *Lactobacillus* spp. and *Bifidobacterium* spp. while increasing pathogenic and non-pathogenic strains of *E. coli* and species from the genus *Clostridium* spp.
Circadian Clock System [50,60]	The circadian rhythm affects the diurnal fluctuations of GM. Stress and changes in the circadian clock system can cause dysregulation of the intestinal microbiota, leading to decreases in Lactobacillus and increases in pathogenic bacteria.
Occupational and Environmental Exposure [50]	Occupational and environmental pollutants, including heavy metals, pesticides, and PAHs can modify GM composition. Shift work and exposure to specific work environments can alter the microbiota, indicating potential health risks.
Diet [50,61]	Diet influences GM diversity and abundance, affecting metabolism and immune responses. Dietary fibers are fermented by GM to produce SCFAs, beneficial for colon health. Variations in diet, such as Mediterranean, ketogenic, vegetarian, or vegan diets, have significant impacts on GM composition.

**Table 2:** Psychobiotics for Schizophrenia.

Psychobiotic	Effect on Schizophrenia	Potential Mechanism
*Lactobacillus rhamnosus* (*JB-1*) [83]	Reduced stress-induced corticosterone levels; decreased depressive behavior	Downregulation of HPA axis activity; alteration in GABA receptor expression
*Mycobacterium vaccae* [7,84]	Reduced anxiety in maze-learning tasks	Not specified
*Bifidobacteria infantis* [7,85]	Increased tryptophan and serotonin levels; decreased pro-inflammatory cytokines	Immunomodulatory effects; modulation of tryptophan metabolism
*Lactobacillus helveticus NS8* [7]	Lowered post-restraint anxiety; enhanced memory; reduced corticosterone and ACTH levels	Anti-inflammatory effects; increase in hippocampal BDNF mRNA
*Lactobacillus rhamnosus JB-1* (additional study) [7]	Elevated concentrations of glutamate, GABA, and tNAA, indicating changes in neural metabolism	Modulation of neurotransmitter concentrations, particularly in excitatory and inhibitory balance
General Probiotics [7]	Observations of reduced gastrointestinal inflammation, immune activation, and modulation of physiological variables including inflammatory markers	Anti-inflammatory properties; vagus nerve stimulation; cytokine modulation

## Abbreviations

CNS	Central Nervous System
MHC	Major Histocompatibility Complex
CD19 and CD20B	Types of Lymphocytes (B Cell Markers)
BDNF	Brain-Derived Neurotrophic Factor
IFNγ	Interferon Gamma
CRP	C-Reactive Protein
IBS	Irritable Bowel Syndrome
NMDAR	N-Methyl-D-Aspartate Receptor
SCFAs	Short-Chain Fatty Acids
GRAS	Generally Recognized As Safe
AI	Artificial Intelligence
DSM-5	Diagnostic and Statistical Manual of Mental Disorders, Fifth Edition
IL-6, IL-8, IL-10, TNF-α	Interleukin 6, Interleukin 8, Interleukin 10, Tumor Necrosis Factor Alpha
HPA Axis	Hypothalamic–Pituitary–Adrenal Axis
ENS	Enteric Nervous System
EGF	Epidermal Growth Factor
GABA	Gamma-Aminobutyric Acid
MGB Axis	Microbiota–Gut–Brain Axis
TLRs	Toll-Like Receptors
LGS	Leaky Gut Syndrome
LPS	Lipopolysaccharide
CCK	Cholecystokinin
PYY	Peptide Tyrosine Tyrosine
GLP-1	Glucagon-Like Peptide-1
CRH	Corticotropin-Releasing Hormone
NO	Nitric Oxide
TPH	Tryptophan Hydroxylase
MAO	Monoamine Oxidase
PET	Positron Emission Tomography
SSRI	Selective Serotonin Reuptake Inhibitors
CLA	Conjugated Linoleic Acid
TSPO	Translocator Protein
